# Effectiveness of Treadmill Training on Balance Control in Elderly People: A Randomized Controlled Clinical Trial

**Published:** 2014-11

**Authors:** Soraya Pirouzi, Ali Reza Motealleh, Fatemeh Fallahzadeh, Mohammad Amin Fallahzadeh

**Affiliations:** 1 Department of Physiotherapy, School of Rehabilitation Sciences, Shiraz University of Medical Sciences, Shiraz, Iran;; 2 School of Medicine, Shiraz University of Medical Sciences, Shiraz, Iran

**Keywords:** Exercise test, Postural balance, Elderly, Walking

## Abstract

Physical exercise would improve postural stability, which is an essential factor in preventing accidental fall among the elderly population. The aim of this study is to examine the effectiveness of treadmill walking on balance improvement among the elderly people.

A total of 30 community dwelling older adults with a Berg Balance Scale score of 36-48 and the ability to walk without aid were considered and divided into control (n=15) and experimental (n=15) groups. Individuals in the experimental group participated in 30 minutes of forward and backward treadmill training based on three times a week interval for a period of four weeks. Individuals in the control group were instructed to continue with their daily routine activity. Before and after training, gait speed was measured by six-minute walk test and balance ability was evaluated by Fullerton Advanced Balance Scale (FABS) and Berg Balance Scale (BBS) tests. Postural sway items such as the Center of Pressure (COP), average displacement and velocity were evaluated by using a force platform system. Data were collected in quiet standing, tandem position and standing on foam pads before and after intervention.

After intervention, balance variables in the experimental group indicated a significant improvement in quiet standing on firm and foam surfaces, but no considerable improvement was shown in tandem position.

A between-group comparison showed a significant reduction in COP velocity in the sagittal plane (P=0.030) during quiet standing and in the frontal plane (P=0.001) during standing on foam, whereas no significant reduction in COP parameters during tandem position was found.

It is recommended that twelve sessions of forward and backward treadmill walk are effective in balance improvement in elderly people.

**Trial Registration Number:** IRCT201209199440N2

## Introduction

Postural control is essential for unassisted movement.^1^ Aging entails physical changes and disrupts the balance control due to the sensory and motor deficits.^2^ About one-third of the elderly population experience accidental fall at least once a year.^1^ Lack of postural stability, misplaced steps, trips and slips are the most common causes of the falls in older adults.^3^

After a fall, elderly people may suffer different injuries such as traumatic spinal cord, brain damage, hip fracture, or even death. In addition to the human suffering, medical cost due to falls has been estimated at about $6.2 billion in 1997 and $7.8 billion in 2002.^4^ Most falls occur during walking whereas the majority of balance training programs merely cover voluntary controlled exercises in a static posture.^5^ Postural mechanisms similar to other motor skills can be improved through training, particularly by perturbation based balance activities.^6^ Several studies have evaluated the effects of perturbation exercises (e.g. simulated slips, unpredictable perturbation^7^ or standing on a movable platform^6^^,^^8^) on balance improvement in older adults. However, the limitations of the above studies were applying the perturbation only for a few sessions and training postural responses while standing but not during walking.

To increase the ability of elderly to control their balance while standing, individuals were challenged by moving base of support and complex walking patterns, to improve voluntary and compensatory postural responses as a fall prevention program.^9^ The aim of this study was to investigate the effectiveness of treadmill training as a dynamic perturbation to challenge postural control reactions in order to improve balance and gait performance.

## Materials and Methods

**Figure 1:**
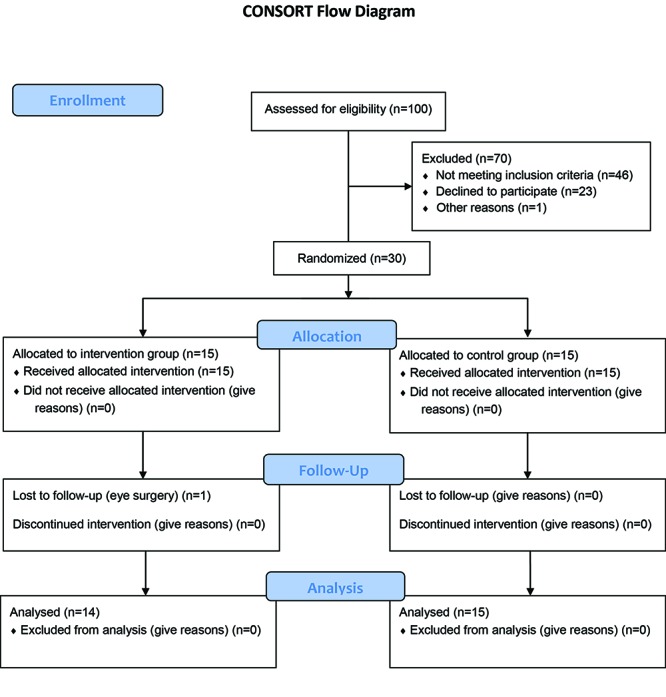
Flowchart showing screening of volunteers and participation in the intervention.

In the screening phase of the study, 100 community dwelling older adults above 60 years of age were considered. Their balance control was evaluated by Fullerton Advanced Balance Scale (FABS) and Berg Balance Scale (BBS) tests. Gait performance was assessed by 6-minute walk test (6MWT). The inclusion criteria were age above 60 years, BBS score between 36 to 48 and the ability to walk on treadmill. The exclusion criteria were: cardiopulmonary diseases, hypertension (≥140/90 mmHg), hypotension (<100/70 mmHg), vertigo, spinal canal stenosis, neurological disorders, severe pain or weakness in the musculoskeletal system, orthopedic problems such as fractures, arthroplasty, the use of walking aids or any other condition that may affect individual’s gait and balance. Amongst the volunteers, 30 individuals met the pre-defined criteria and were equally divided into experimental and control groups according to blocked randomization (figure 1). After withdrawal of one person from the program, 14 participants with the age range of 61-82 years (70.64±5.68) and male to female ratio of 0.4 completed the training as the experimental group. In parallel, 15 persons with the age range of 62-81 years (71.40±6.12) and male to female ratio of 6.6 completed the training as the control group.

Each individual in the experimental group performed 30 minutes of the treadmill training based on three times a week interval for a period of four weeks (12 sessions). They were instructed to walk on treadmill (Proteus IMT-8000/8500) on a self-selected comfortable speed. Each training session included 5-minute warm-up, 10-minute forward walking, 10-minute backward walking and 5-minute cool-down exercises. For safety reasons, a harness was deployed during walks. The individuals in the control group continued with their normal daily activity.

The balance performance of all individuals in the experimental group was assessed before and after 4-week treadmill training. Likewise, the balance performance of the control group was assessed before and after 4-week daily routine activity. Postural sway in frontal and sagittal plane was measured by a Kistler-9386AA force platform. Individuals were instructed to remain stable while performing the tests on the force plate. They were instructed to position their feet shoulder width apart from each other and holding arms by their sides while looking straight ahead to a fixed point at 4.5-meter distance on an adjustable board aligned with eye level. Nine trials in three random ordered conditions were conducted, three trials on quiet standing, three trials on tandem position and three trials while standing on foam pads. Each trial lasted 30 seconds with data acquisition rate of 120 Hz. The signals were then filtered at 6 Hz.

*Statistical Analysis*

SPSS software version 15 was used for data analysis. Independent sample t-test was used to compare the pre and post training values between the groups. Before and after training, gait speed and balance variables in each group were analyzed by paired *t* test. A significant level of 0.05 was set for all analyses.

## Results

There were no significant differences between the two groups in demographic data and balance variables. As shown in table 1, 14 individuals in the experimental and 15 in the control groups completed the study. BBS demonstrated 8.14 points, FABS showed 35%, and 6MWT exhibited 12% improvement in the experimental group with respect to the control.

**Table 1:** Gait speed and balance scales in experimental and control groups

**Tests**	**Experimental group (n=14)**			**Control group (n=15)**		
	**Pre intervention **	**Post intervention **	**P value**	**Pre intervention **	**Post intervention **	**P value**
BBS	41.64±3.100	49.78±3.19	0.001	42.73±4.39	42.80±4.21	0.843
FABS	24.95±3.62	33.71±3.79	0.001	25.80±4.36	25.80±4.75	1.000
6MWT	268.75±80.46	300.14±85.89	0.001	272.13±79.47	275.06±80.11	0.085

BBS: Berg balance scale; FABS: Fullerton advance balance scale; 6MWT: Six-minute walking test

According to table 2, a significant reduction of COP velocity was shown in the sagittal plane during quiet standing and in the frontal plane during standing on foam between the groups. There was no significant reduction in balance variables during tandem standing between groups.

**Table 2:** Balance variables in quiet, tandem and standing on foam between groups

	**Variable**	**Pre intervention value**			**Post intervention value**		
		**Experimental **	**Control **	**P value**	**Experimental**	**Control**	**P value**
Quiet Standing	DIS AP	3.19±0.88	3.10±0.77	0.768	2.77±0.75	2.90±1.17	0.720
	DIS ML	2.98±1.25	2.88±1.06	0.823	2.04±0.78	2.71±1.5	0.083
	VEL AP	5.92±2.20	6.66±1.76	0.324	5.19±1.25	6.47±1.69	0.030
	VEL ML	6.20±2.53	5.88±2.50	0.736	4.59±1.25	5.91±2.83	0.153
	VEL T	9.53±3.51	9.91±2.97	0.752	7.69±2.46	9.82±3.28	0.059
Tandem standing	DIS AP	4.15±2.47	2.99±1.07	0.109	2.73±1.25	3.55±1.43	0.096
	DIS ML	5.20±1.16	4.47±1.64	0.180	4.19±0.71	5.07±1.0	0.874
	VEL AP	14.35±6.43	15.07±6.47	0.767	11.37±2.40	14.36±6.07	0.097
	VEL ML	18.75±6.01	17.54±5.64	0.579	16.77±3.55	16.49±3.79	0.825
	VEL T	26.11±4.43	25.57±8.91	0.875	22.20±4.34	24.29±7.47	0.370
Foam standing	DIS AP	6.89±1.29	6.25±1.22	0.189	6.08±0.95	6.78±1.87	0.218
	DIS ML	6.43±2.29	5.29±1.87	0.152	5.22±1.40	6.25±2.24	0.154
	VEL AP	20.04±5.82	18.07±3.47	0.355	18.45±5.45	19.24±5.58	0.702
	VEL ML	17.63±5.35	13.45±4.23	0.270	13.44±3.61	14.70±5.13	0.001
	VEL T	29.63±8.26	24.86±6.67	0.106	25.19±6.78	2676±7.37	0.555

DIS AP: Displacement of COP in Anteroposterior (sagittal) plane; DIS ML: Displacement of COP in Mediolateral (frontal) plane; VEL AP: Velocity of COP in Anteroposterior plane; VEL ML: Velocity of COP in Mediolateral plane; VEL T: Total velocity

Based on table 3, after intervention during quiet standing, participants in the experimental group showed a significant reduction in COP displacement in both frontal and sagittal planes, as well as in COP velocity in the frontal plane and total velocity. However, there was no significant reduction in balance variables during tandem standing in the experimental group. Furthermore, participants **in the experimental group during standing on foam had a significant reduction in all balance variables.**

**Table 3:** Balance variables in quiet, tandem and standing on foam inter groups

	**Variable**	**Experimental**			**Control **		
		**Pre intervention **	**Post intervention **	**P value**	**Pre intervention **	**Post intervention **	**P value**
Quiet Standing	DIS AP	3.19±0.88	2.77±0.75	0.030	3.10±0.77	2.90±1.17	0.478
	DIS ML	2.98±1.25	2.04±0.78	0.017	2.88±1.06	2.71±1.50	0.385
	VEL AP	5.92±2.20	5.19±1.25	0.191	6.66±1.76	6.47±1.69	0.686
	VEL ML	6.20±2.53	4.59±1.25	0.036	5.88±2.50	5.91±2.83	0.973
	VEL T	9.53±3.51	7.69±2.46	0.005	9.91±2.97	9.82±3.28	0.919
Tandem standing	DIS AP	4.15±2.47	2.73±1.25	0.095	2.99±1.07	3.55±1.43	0.020
	DIS ML	5.20±1.16	4.19±0.71	0.452	4.47±1.64	5.07±1.00	0.067
	VEL AP	14.35±6.43	11.37±2.40	0.061	15.07±6.47	14.36±6.07	0.650
	VEL ML	18.75±6.01	16.77±3.55	0.101	17.54±5.64	16.49±3.79	0.437
	VEL T	26.11±4.43	22.20±4.34	0.065	25.57±8.91	24.29±7.47	0.540
Foam standing	DIS AP	6.89±1.29	6.08±0.95	0.006	6.25±1.22	6.78±1.87	0.111
	DIS ML	6.43±2.29	5.22±1.40	0.030	5.29±1.87	6.25±2.24	0.002
	VEL AP	20.04±5.82	18.45±5.45	0.012	18.07±3.47	19.24±5.58	0.243
	VEL ML	17.63±5.35	13.44±3.61	0.008	13.45±4.23	14.70±5.13	0.053
	VEL T	29.63±8.26	25.19±6.78	0.001	24.86±6.67	2676±7.37	0.100

DIS AP: Displacement of COP in Anteroposterior (sagittal) plane; DIS ML: Displacement of COP in Mediolateral (frontal) plane; VEL AP: Velocity of COP in Anteroposterior plane; VEL ML: Velocity of COP in Mediolateral plane; VEL T: Total velocity

## Discussion

The present study demonstrated that twelve sessions of treadmill training improve balance performance and gait speed among the elderly people.

A significant improvement of 8.14 points in BBS and 35% in FABS indicated that our exercise program was efficient in improving balance performance.10 These findings are in line with a previous study that reported 13.8% improvement in walking speed after three weeks of training.11 According to movement studies, the human motor system can react rapidly to unexpected conditions and is able to produce predictive adjustments in response to repeated perturbations. In addition, adaptive mechanisms necessary for coordination patterns, body orientation, and balance stability can be improved by treadmill walking.^12^Furthermore, during backward treadmill walking, gait velocity, stride length and duration of swing phase decreases whereas double support phase increases. This process activates the soleus reflex that is likely part of the motor program controlling the backward walking.^13^

In addition, walking on a treadmill is more effective in activating the central gait pattern generator and enhances motor learning than other type of exercises. The results of the present study are in agreement with Lau et al. reporting that 10 sessions of speed-dependent treadmill training is effective in improving balance in subacute stroke individuals.^14^

In the present study, a significant reduction in COP average displacement and velocity in the experimental group suggests that treadmill training is effective in improving postural control among the elderly population. Our findings are in line with the study of Fernanda et al.^15^ indicating a significant reduction in COP displacement and velocity values in elderly women.

Inherent to the aging process, functional and structural changes such as abnormal visual, vestibular and sensory integration occur, which leads to decreased postural stability and increased risk of accidental fall.^14^ Sensory integration can be improved through specific training such as altering the standing surface or its movement, in order to enhance postural stability in elderly people.^16^ During forward walk on treadmill, somatosensory information is distorted whereas during backward walk visual sensation is additionally distorted. According to the sensory re-weighting theory, individuals mainly use vestibular input to maintain balance. This might be a reason for balance recovery in the present study.

In addition, treadmill walking has a clear-cut effect on posture. After forward and backward walking, forward and backward inclination occurs^12^ that leads to anterior and posterior muscle stretching. This is followed by co-contraction of muscles which maintains balance.^17^

Our results are in agreement with findings of Shimada et al. who investigated the effects of perturbed walking exercise using a bilateral separated treadmill in frail elderly people. In spite of improvement in balance, no significant differences were seen in the time of the first accidental fall.^18^

The findings of our experiment correspond with another study that investigated the efficacy of the perturbation based balance training (PBBT) on time to stabilization in older adults. Their findings indicated that time to stabilization of COP was 41.6 shorter than baseline test, and they concluded that PBBT is effective on postural stability in older adults.^6^ Also, a study by Salsabili et al. on diabetic people showed a similar result, however, they tried to stimulate the ankle and hip strategy through small movement on a platform.^8^ It differs from our study that attempted to stimulate step strategy as a compensatory factor to improve balance.^3^

There are some reports that differ with the current study, mostly because of differences in the nature of the exercises. For instance, one study revealed that under experimental conditions, strenuous treadmill exercise results in increased body sway in healthy young individuals. Their aim was to show the effects of fatigue on postural stability.^19^

The performance in tandem position for elderly people is more challenging and deteriorates with age. During this position, the stance width decreases, but the length of the support phase increases. Tibialis anterior has an important role in ankle stiffness by pressing the lateral side of the foot during stance and control of balance in the frontal plane.^20^ The level of force variability in the tibialis anterior muscle, in static phase is higher than the dynamic phase in elderly. This may justify the difficulties in maintaining balance during the dynamic phase of tandem stance in older adults. Our findings showed that treadmill training was not effective enough to reduce the COP average displacement and velocity significantly in tandem standing position. This might be due to differences between tandem and quiet standing. Additionally, it might be more effective with longer duration of training.

The main limitations of this study were short duration of follow-up and small sample size. The implications of these findings for future research could be the evaluation of balance improvement after different perturbation activities and longer follow-up. 

## Conclusion

Combination of forward and backward walking on a treadmill is an effective approach to improve postural control and gait performance in the elderly population. 
